# Supplementary material for: Microbial genetic and transcriptional contributions to oxalate degradation by the gut microbiota in health and disease
Source: eLife. 2021 Mar 26;10:e63642. doi: 10.7554/eLife.63642 (PMC8062136; doi:10.7554/eLife.63642)
Supplement: Supplementary file 1. — (a) Description of populations used in the present study. (b) Population-level contribution of species to metagenomic or metatranscriptomic OXC, in healthy, ulcerative colitis (UC), or Crohn’s disease (CD) patients. (c) Population-level contribution of species to metagenomic or metatranscriptomic FRC, in healthy, UC, or CD patients. (d) Taxonomic contributions to frc or oxc genes* inferred by BURRITO in 2359 metagenomic samples (see Materials and methods for detailed description). (e) The dominant taxa detected by 16S rRNA sequencing in the baseline fecal samples (n = 17) from the mouse study in Figure 6. [file elife-63642-supp1.docx]

**Supplementary file 1a. Description of populations used in the present study.**

**Supplementary file 1b. Population-level contribution of species to metagenomic or metatranscriptomic OXC, in healthy, Ulcerative colitis (UC) or Crohn's Disease (CD) patients**

| Species | Metagenome | | | Metatranscriptome | | |
| --- | --- | --- | --- | --- | --- | --- |
|  | Healthy | UC | CD | Healthy | UC | CD |
| *Escherichia coli* | 36.14 | 63.04 | 64.83 | 6.92 | 31.66 | 23.86 |
| *Oxalobacter formigenes* | 16.70 | 1.13 | 2.74 | 62.58 | 23.87 | 28.38 |
| *Azospirillum sp.* | 9.06 | 0.12 | 1.52 | 0.04 | ND | 0.73 |
| *Bifidobacterium pseudocatenulatum* | 8.94 | 7.15 | 10.36 | ND | 2.39 | 5.38 |
| *Muribaculaceae bacterium* | 4.58 | 1.51 | 0.75 | 5.61 | 3.52 | 1.11 |
| *Porphyromonadaceae bacterium* | 4.42 | 2.29 | 1.46 | 4.51 | 6.83 | 0.75 |
| *Bacteroidales bacterium* | 3.36 | 3.58 | 3.00 | 2.72 | 4.40 | 6.17 |
| *Prevotella sp.* | 3.04 | 0.13 | 0.23 | 1.18 | ND | 0.07 |
| *Bifidobacterium dentium* | 2.83 | 2.70 | 3.72 | 0.29 | ND | 2.82 |
| *Bifidobacterium animalis* | 2.52 | 5.80 | 1.32 | 2.02 | 12.10 | 6.01 |
| *Enterorhabdus caecimuris* | 1.08 | 0.25 | 0.10 | 2.59 | ND | ND |
| *Lactobacillus gasseri* | 0.80 | 0.99 | 1.19 | 0.04 | ND | 0.10 |
| *Lactobacillus acidophilus* | 0.69 | 1.23 | 1.60 | 0.74 | 1.51 | 8.60 |
| *Bifidobacterium sp.* | 0.66 | 0.77 | 0.55 | ND | ND | ND |
| *Lactobacillus reuteri* | 0.41 | 0.06 | 0.22 | 1.63 | 3.09 | 3.60 |
| *Bifidobacterium pseudolongum* | 0.32 | 0.97 | 0.24 | 0.55 | 4.45 | 0.30 |
| *Bifidobacterium cuniculi* | 0.29 | 0.37 | 0.29 | ND | 1.42 | 0.05 |
| *Lactobacillus mucosae* | 0.25 | ND | 0.05 | ND | ND | 0.02 |
| *Rhodospirillaceae bacterium* | 0.25 | 0.04 | 0.26 | 4.82 | 0.96 | 1.34 |
| *Lactobacillus amylovorus* | 0.23 | 1.01 | 2.10 | 0.30 | 1.25 | 4.32 |
| *Bifidobacterium choerinum* | 0.19 | 0.72 | 0.37 | ND | 1.70 | 3.84 |
| *Bifidobacterium magnum* | 0.14 | 2.73 | 0.04 | 0.07 | ND | ND |
| *Bifidobacterium criceti* | 0.00 | ND | ND | ND | ND | ND |
| Others | 3.12 | 3.42 | 3.05 | 3.40 | 0.86 | 2.54 |

ND: Not detected

**Supplementary file 1c. Population-level contribution of species to metagenomic or metatranscriptomic FRC, in healthy, Ulcerative colitis (UC) or Crohn's Disease (CD) patients**

| Species | Metagenome | | | Metatranscriptome | | |
| --- | --- | --- | --- | --- | --- | --- |
|  | Healthy | UC | CD | Healthy | UC | CD |
| *Escherichia coli* | 36.14 | 63.04 | 64.83 | 6.92 | 31.66 | 23.86 |
| *Oxalobacter formigenes* | 16.70 | 1.13 | 2.74 | 62.58 | 23.87 | 28.38 |
| *Azospirillum sp.* | 9.06 | 0.12 | 1.52 | 0.04 | ND | 0.73 |
| *Bifidobacterium pseudocatenulatum* | 8.94 | 7.15 | 10.36 | ND | 2.39 | 5.38 |
| *Muribaculaceae bacterium* | 4.58 | 1.51 | 0.75 | 5.61 | 3.52 | 1.11 |
| *Porphyromonadaceae bacterium* | 4.42 | 2.29 | 1.46 | 4.51 | 6.83 | 0.75 |
| *Bacteroidales bacterium* | 3.36 | 3.58 | 3.00 | 2.72 | 4.40 | 6.17 |
| *Prevotella sp.* | 3.04 | 0.13 | 0.23 | 1.18 | ND | 0.07 |
| *Bifidobacterium dentium* | 2.83 | 2.70 | 3.72 | 0.29 | ND | 2.82 |
| *Bifidobacterium animalis* | 2.52 | 5.80 | 1.32 | 2.02 | 12.10 | 6.01 |
| *Enterorhabdus caecimuris* | 1.08 | 0.25 | 0.10 | 2.59 | ND | ND |
| *Lactobacillus gasseri* | 0.80 | 0.99 | 1.19 | 0.04 | ND | 0.10 |
| *Lactobacillus acidophilus* | 0.69 | 1.23 | 1.60 | 0.74 | 1.51 | 8.60 |
| *Bifidobacterium sp.* | 0.66 | 0.77 | 0.55 | ND | ND | ND |
| *Lactobacillus reuteri* | 0.41 | 0.06 | 0.22 | 1.63 | 3.09 | 3.60 |
| *Bifidobacterium pseudolongum* | 0.32 | 0.97 | 0.24 | 0.55 | 4.45 | 0.30 |
| *Bifidobacterium cuniculi* | 0.29 | 0.37 | 0.29 | ND | 1.42 | 0.05 |
| *Lactobacillus mucosae* | 0.25 | ND | 0.05 | ND | ND | 0.02 |
| *Rhodospirillaceae bacterium* | 0.25 | 0.04 | 0.26 | 4.82 | 0.96 | 1.34 |
| *Lactobacillus amylovorus* | 0.23 | 1.01 | 2.10 | 0.30 | 1.25 | 4.32 |
| *Bifidobacterium choerinum* | 0.19 | 0.72 | 0.37 | ND | 1.70 | 3.84 |
| *Bifidobacterium magnum* | 0.14 | 2.73 | 0.04 | 0.07 | ND | ND |
| *Bifidobacterium criceti* | 0.00 | ND | ND | ND | ND | ND |
| Others | 3.12 | 3.42 | 3.05 | 3.40 | 0.86 | 2.54 |

ND: Not detected

**Supplementary file 1d. Taxonomic contributions to *frc* or *oxc* genes* inferred by BURRITO in 2359 metagenomic samples (See Methods for detailed description).**

| Species | Population-level contribution (%) | Contribution (%) among positive samples (mean±S.D.) |
| --- | --- | --- |
| *Escherichia coli* | 55.02 | 75.51 ± 33.42 |
| *Oxalobacter formigenes* | 17.54 | 53.24 ± 40.85 |
| *Bifidobacterium pseudocatenulatum* | 12.87 | 60.6 ± 37.96 |
| *Klebsiella pneumoniae* | 5.85 | 46.36 ± 37.67 |
| *Bifidobacterium animalis* | 4.58 | 43.89 ± 40.73 |
| *Bifidobacterium dentium* | 1.85 | 20.24 ± 30.09 |
| *Klebsiella oxytoca* | 1.02 | 31.2 ± 37.08 |
| *Lactobacillus gasseri* | 0.69 | 23.73 ± 35.3 |
| *Lactobacillus reuteri* | 0.43 | 52.35 ± 40.65 |
| *Lactobacillus animalis* | 0.1 | 52.39 ± 67.34 |
| *Hafnia alvei* | 0.06 | 5.12 ± 6.75 |

**For genomic content input for BURRITO, we assumed 1 copy of frc and oxc for each genome to minimize bias, as such information is not available for all species (see methods for details). Thus, taxonomic contributions to both genes are the same.*

**Supplementary file 1e. The dominant taxa detected by 16S rRNA sequencing in the baseline fecal samples (n=17) from the mouse study in Fig. 6.**

| **Family** | **Genus** | **Prevalence** | **Mean relative**  **Abundance** |
| --- | --- | --- | --- |
| *Lachnospiraceae* | *Blautia* | 100% | 0.48 |
| *Peptostreptococcaceae* | *Clostridioides* | 100% | 0.36 |
| *[Eubacterium]*  *coprostanoligenes_group* | *[Eubacterium]*  *coprostanoligenes_group* | 100% | 0.02 |
| *Erysipelotrichaceae* | *[Clostridium]_innocuum_group* | 100% | 0.03 |
| *Lachnospiraceae* | *Anaerostipes* | 100% | 0.01 |
| *Lachnospiraceae* | *Lachnoclostridium* | 100% | 0.02 |
| *Oscillospiraceae* | *Flavonifractor* | 100% | 0.02 |
| *Lachnospiraceae* | *Hungatella* | 94% | 0.03 |
| *Peptostreptococcaceae* | *unclassified* | 71% | <0.1 |
| *Lachnospiraceae* | *unclassified* | 65% | <0.1 |
