# Supplementary material for: Microbial genetic and transcriptional contributions to oxalate degradation by the gut microbiota in health and disease
Source: eLife. 2021 Mar 26;10:e63642. doi: 10.7554/eLife.63642 (PMC8062136; doi:10.7554/eLife.63642)
Supplement: Transparent reporting form [file elife-63642-transrepform.docx]

***eLife’s* transparent reporting form**

We encourage authors to provide detailed information *within their submission* to facilitate the interpretation and replication of experiments. Authors can upload supporting documentation to indicate the use of appropriate reporting guidelines for health-related research (see [EQUATOR Network](http://www.equator-network.org/%20)), life science research (see the [BioSharing Information Resource](https://biosharing.org/)), or the [ARRIVE guidelines](http://www.plosbiology.org/article/info:doi/10.1371/journal.pbio.1000412) for reporting work involving animal research. Where applicable, authors should refer to any relevant reporting standards documents in this form.

If you have any questions, please consult our Journal Policies and/or contact us: [editorial@elifesciences.org](mailto:editorial@elifesciences.org).

**Sample-size estimation**

Please outline where this information can be found within the submission (e.g., sections or figure legends), or explain why this information doesn’t apply to your submission:

We did not perform an explicit power analysis since there is no previous data on our animal model and experimental conditions. We thought that 5-6 mice per group is a reasonable number of mice per group to show a significant change in urinary oxalate. We performed the 5-6 biological replicates per each mouse group. This information is in our methods section on page 35.

**Replicates**

Please outline where this information can be found within the submission (e.g., sections or figure legends), or explain why this information doesn’t apply to your submission:

Biological replicates are parallel measurements of biologically distinct samples that capture random biological variation, which may itself be a subject of study or a noise source. Technical replicates are repeated measurements of the same sample that represent independent measures of the random noise associated with protocols or equipment. In each mouse group, we performed 5-6 biological replicates by gavaging material (culture media, growing culture of strain OXCC13 or PH1) into 5-6 mice.

Technical replicates were performed for measurements. Urinary oxalate and creatinine, fecal oxalate, qPCR, and fecal oxalate were measured in technical duplicates. This information is in our methods section on page 35.

**Statistical reporting**

- Statistical analysis methods should be described and justified
- Raw data should be presented in figures whenever informative to do so (typically when N per group is less than 10)
- For each experiment, you should identify the statistical tests used, exact values of N, definitions of center, methods of multiple test correction, and dispersion and precision measures (e.g., mean, median, SD, SEM, confidence intervals; and, for the major substantive results, a measure of effect size (e.g., Pearson's r, Cohen's d)
- Report exact p-values wherever possible alongside the summary statistics and 95% confidence intervals. These should be reported for all key questions and not only when the p-value is less than 0.05.

Please outline where this information can be found within the submission (e.g., sections or figure legends), or explain why this information doesn’t apply to your submission:

Statistical methods can be found in the corresponding figure legends, or in results text if no figures were associated with.

(For large datasets, or papers with a very large number of statistical tests, you may upload a single table file with tests, Ns, etc., with reference to sections in the manuscript.)

**Group allocation**

- Indicate how samples were allocated into experimental groups (in the case of clinical studies, please specify allocation to treatment method); if randomization was used, please also state if restricted randomization was applied
- Indicate if masking was used during group allocation, data collection and/or data analysis

Please outline where this information can be found within the submission (e.g., sections or figure legends), or explain why this information doesn’t apply to your submission:

The group allocation details for the *O. formigenes* colonization experiment can be found in Results and Methods sections.

**Additional data files (“source data”)**

- We encourage you to upload relevant additional data files, such as numerical data that are represented as a graph in a figure, or as a summary table
- Where provided, these should be in the most useful format, and they can be uploaded as “Source data” files linked to a main figure or table
- Include model definition files including the full list of parameters used
- Include code used for data analysis (e.g., R, MatLab)
- Avoid stating that data files are “available upon request”

Please indicate the figures or tables for which source data files have been provided:

Figures 2-6
